# The Outcome of Cell Therapy Treating Urinary Incontinence Correlates with Precise Cell Localization in the Sphincter Complex

**DOI:** 10.3390/biomedicines13040917

**Published:** 2025-04-09

**Authors:** Niklas Harland, Liv Johnen, Kamal T. Avula, Andrea Buzanich-Ladinig, Lukas Schwarz, Jasmin Knoll, Arnulf Stenzl, Wilhelm K. Aicher

**Affiliations:** 1 Center for Medical Research, University of Tuebingen Hospital, 72072 Tuebingen, Germany; niklas.harland@med.uni-tuebingen.de (N.H.); liv.johnen@student.uni-tuebingen.de (L.J.); kamal.avula@student.reutlingen-university.de (K.T.A.); uro@stenzl.net (A.S.); 2 Clinical Centre for Population Medicine in Fish, Pig and Poultry, Clinical Department for Farm Animals and Food System Science, University of Veterinary Medicine Vienna, 1210 Vienna, Austria; andrea.ladinig@vetmeduni.ac.at (A.B.-L.); lukas.schwarz@vetmeduni.ac.at (L.S.); 3 Department of Cardio-Thoracic Surgery, University of Tuebingen Hospital, 72076 Tuebingen, Germany

**Keywords:** stress urinary incontinence, cell therapy, cell injection, muscle regeneration, myogenic progenitor cell, adipose tissue-derived stromal cell, large animal model of incontinence

## Abstract

**Background/Objectives**: Urethral sphincter muscle deficiency is the leading cause of stress urinary incontinence. Preclinical and clinical studies suggested that cell therapy may improve the situation. However, the overall efficacy of cell therapies did often not satisfy the patient’s needs. We, therefore, investigated in a large animal model of incontinence if the localization of injected regenerative cells in the deficient urethral sphincter muscle correlated with the outcome. **Methods**: Urethral sphincter insufficiency was induced in three cohorts of pigs and confirmed by urodynamics. Then, either myogenic progenitor cells (MPCs) or adipose tissue-derived stromal cells (ADSCs) were injected into the injured sphincter complex by Williams needle under visual using a cystoscope. Sham-treated animals served as controls. Functional sphincter muscle regeneration was monitored by urodynamics over 5 weeks of follow-up. The localization of the injected cells was investigated by histology of cryosections of the tissue targeted. **Results**: Injection of MPCs near the sphincter muscle yielded better functional recovery when compared to MPC injections in adjacent sides. By contrast, injection of ADSCs in the submucosal tissue adjacent to the muscle led to better regeneration when compared to ADSC injections into the sphincter muscle. After five weeks of follow-up, MPCs yielded an overall robust but not significant improvement when compared to mock-treated controls, while ADSC injections reached significance. **Conclusions**: This small proof-of-principle study suggests that the clinical outcome of cell therapy for urinary incontinence depends on the choice of therapeutic cells and the precise localization of the cells in the tissue targeted as well.

## 1. Introduction

Stress urinary incontinence (SUI) is a significant burden to the individual affected, to the health professionals involved, and to the health care systems [1,2]. For women, the prevalence of suffering from any form of urinary incontinence (UI) was computed at approximately 45% [3], and medical records and surveys suggested that about 30% of them suffer from SUI [4,5,6,7]. In men, UI is reported in 10% to more than 30% of the adult population, depending on age and health conditions [8]. Incontinence contributes to different comorbidities, including urinary infections, dermal irritations, and local inflammation [9]. The aetiology of SUI includes an insufficiency of the urethral sphincter muscle function or a total loss of it [10]. In women, SUI is associated with mechanical stress to the lower pelvic floor by pregnancy and vaginal delivery, as well as with loss of tissue elasticity during hormonal changes in the menopause [6,7]. In men, SUI may develop upon prostate surgeries [6,10]. Patients suffering from mild forms of SUI may manage the sequela of SUI by adapting their day-to-day routine, fluid uptake, and discharge. Individuals suffering from moderate to severe SUI require treatment. This includes physical exercise of the lower pelvic floor muscles, electromagnetic stimulations, and a pharmacological regimen [11,12,13,14]. Recent experiments in rodents suggested that ultrasound stimulation of the lower pelvic floor could activate muscle stem cells and thus ameliorate SUI in this model [15]. If, however, in some cases, non-surgical treatments fail to yield satisfactory or lasting results, different surgical options are at hand [10]. Based on the finding that loss of functional muscle tissue is associated with SUI [16], urologists hypothesised that injection of active components supporting local muscle regeneration in the sphincter complex may ameliorate or even cure SUI [17,18]. Injection of myogenic progenitor cells (MPCs) was considered one possibility to strengthen the sphincter muscle [19,20,21,22]. In this case, the MPCs would align to the sphincter muscle, differentiate to generate myofibers, and integrate [21]. In addition, mesenchymal stromal cells, including adipose tissue-derived stromal cells (ADSCs), were considered a potent regimen for SUI treatment, as they have been shown to support wound healing, tissue regeneration, vascularization, and modulate immune responses by the release of exosomes and/or secretion of cytokines and growth factors [15,23,24,25,26,27,28]. This hypothesis was also investigated in several feasibility trials [15,17,26,29,30,31,32,33,34]. However, some studies failed to meet the expectations [35], quite a few studies included only small cohorts and short follow-up times, and a few publications even had to be retracted. Studies on the cost-effectiveness of SUI treatment favoured cell therapy over sling surgery [36]. Likewise, many preclinical animal models of SUI cell therapy were very promising [37,38]. Yet, the outcome of many reports encouraged additional pre-clinical and clinical studies to investigate the surgical and biological details of SUI cell therapy [38].

Recently, we developed a large animal model of SUI to investigate the efficacy of cell therapy [38,39,40,41]. Injections of ADSCs yielded a significant and complete regeneration of the urethral muscle within 5 weeks of follow-up (100%, n = 6 pigs, *p* < 0.05) in comparison to the sham controls (67%, n = 6 pigs), while injection of MPCs improved the urethral muscle to a level of 81.5% (5 pigs, not significant above sham controls) [41]. After the sacrifice of the animals, the sphincter complex of two representative animals from each of the three experimental groups—(i) sham controls, (ii) MPC-, and (iii) ADSC-treated pigs—were investigated by histology. The results suggested that not only the type of cell injected but also the exact localization of the cell applied may influence the clinical outcome of cell therapy [41].

In this study, we, therefore, investigated the exact localization of the MPCs and ADSCs in the urethral sphincter complex in all animals of our recent SUI cell therapy study [41]. Our study suggests that injections of MPCs yielded better outcomes after injection *into* the sphincter muscle or nearby, while MPC injections in the mucosal layer or at remote sides of the urethra brought forth less tissue regeneration. By contrast, injection of ADSCs in the mucosa *close* to the urethral muscle layer granted better muscle regeneration when compared to injections in the muscle or at remote sides. Loss of cells, of course, did not facilitate muscle regeneration above levels of sham controls. We conclude that urethral muscle regeneration by cell therapy depends on the type of cells applied. In addition, the outcome is also influenced by injecting the corresponding cells into the optimal location for improved tissue repair.

## 2. Materials and Methods

### 2.1. Setting Up a Large Animal Model of Stress Urinary Incontinence

Eighteen female German landrace pigs (=gilts) were obtained from a breeder with excellent health status and brought one week before surgery to the animal husbandry facilities for adaptation. The animals were maintained with food and water supply ad libitum. Twenty-four hours before surgery, pigs were set on a low-food diet. Immediately before surgery, all animals were sedated (0.05 mg/kg Atropin, 4 mg/kg Azaperon i.m.) and then anaesthetized (4 mg/kg Propofol i.v., Fentanyl 70 μg/kg/h, Isoflurane 1 Vol% under controlled artificial respiration) as described [40]. In deep anaesthesia, the localization of the urethral sphincter muscle was determined by measuring the urethral wall pressure by urodynamics alongside the urethra (i.e., urethral pressure profilometry, Aquarius TT^®^, UDS120 software V12; Laborie, Mississauga, ON, Canada) in untreated pigs as described [40]. To this end, a sensor catheter was inserted into the bladder, air-charged and pulled out slowly (Dual-Sensor Catheter; T-Doc, Laborie, Williston, VT, USA). Both, the vesicular and the urethral wall pressures were relayed from the sensors in the catheter to the urodynamic device and computed (Nexam Pro WPU-L4; Laborie). The urethral wall pressure curve was displayed as a histogram, and the histogram profile was used to localise the sphincter complex in each pig [38,40]. This value determined the initial wall pressure. It was set to 100% in each pig as the pre-treatment reference (“pre”). Then, sphincter insufficiency was induced surgically by high-frequency electro-dissection (HF-DE; i.e., electrocautery) of the sphincter muscle (Vio300 D, monopolar cut, effect 4, maximal power 100 W; ERBE Elektromedizin, Tübingen, Germany) and balloon dilatation of the urethra, followed by urodynamics to determine the success of SUI induction (=“post”) [38,40]. On day 3 after SUI induction, pigs were anaesthetized to corroborate the efficacy of the SUI induction by urodynamics. Urine samples were collected before each intervention and then weekly during follow-up to monitor irregularities. The animal study was approved by the State of Baden-Württemberg Animal Welfare authorities (Regierungspräsidium Tübingen, approval date: 28 July 2020) under file number 35/9185.81-2, CU01-20G and performed under full compliance with the ARRIVE 2.0 standards and all other relevant regulations.

### 2.2. Production of Homologous Porcine Cells for Injection

To avoid rejection of the injected cells in the gilts, homologous MPCs were isolated aseptically from *Musculus semitendinosus* of male littermates from cohort 2 pigs. Homologous ADSCs were aseptically isolated from the subcutaneous fat tissue of male littermates of cohort 3 pigs. The porcine male MPCs and ADSCs were expanded following optimised protocols [41,42,43,44,45]. In brief, for MPC production, muscle tissue was minced by blades and enzymatically degraded in HBSS complemented by protease mix (0.2% collagenase I, 0.025% trypsin, 0.001 DNase 1) for 20 min at 37 °C. Debris was sedimented by cooling the sample on wet ice for 5 min. The supernatant was diluted 1:1 with MEMα, and the cells were separated from debris by filtration through a 100 μm cell strainer. The cells were sedimented by centrifugation (800× *g*, 10 min, 4 °C), washed by MEMα, and incubated for 20 min at 37 °C in protease mix again. Debris was removed by passing the cells through a cell strainer again. The MPCs were then enriched by a Percoll^®^-gradient centrifugation (15,000× *g*, 10 min, 4 °C. Sigma-Aldrich, Taufkirchen, Germany). The interface containing MPCs was collected, and the cells were washed by MEMα and centrifugation (800× *g*, 10 min, 4 °C), seeded in type I collagen-coated flasks, and expanded in MPC expansion medium (F10 medium, complemented by 15% heat-inactivated FBS, 5 ng/mL bFGF, glutamine, and antibiotics [42,43,44]. The expression patterns of myogenic markers, differentiation capacity and electrophysiologic competence of the MPCs employed have recently been published [44]. For ADSC production, subcutaneous fat was isolated aseptically, and visible blood vessels and connective tissue were removed [45]. The fat was minced by blades, and the tissue pieces were incubated in collagenase buffer (0.1% collagenase 1, 1% BSA in PBS, 37 °C, 30 min). The proteolysis was stopped by adding 1 volume ADSC expansion medium (DMEM low glucose, 10% heat-inactivated FBS, 5 mM HEPES, glutamine, antibiotics). To sediment debris and to facilitate the floating up of adipocytes, the blend is incubated for 10 min at ambient temperature. The remaining fat and adipocytes were aspirated from the top, and the ADSCs were enriched by passing the liquid through a 100 μm cell strainer, followed by centrifugation. The cell pellet was washed twice in F10 medium by centrifugation, and the cells are expanded in the ADSC expansion medium [45]. The expression patterns of stromal cell markers, cytokines, and the differentiation capacity of the ADSCs employed have recently been published [41,44].

### 2.3. Transurethral Cell Injection and Follow-Up by Urodynamics

Six gilts of cohort 1 (=sham controls) were anaesthetized three days after induction of sphincter deficiency to confirm a sufficient induction of incontinence by urodynamics. Cohort 1 animals were not injected with cells or fluorescent microparticles (fMP), cohort 2 animals were treated with MPCs plus fMPs, and cohort 3 animals with ADSCs plus fMPs. Before cell injection on day 3, sufficient sphincter deficiency was confirmed in the six gilts of cohorts 2 and 3, respectively. Then, in cohort 2, 6 × 10^5^ homologous MPCs plus 6 × 10^5^ FITC-labelled fMPs each were injected in two aliquots sidewise into the sphincter complex by a modified Williams Cystoscopic Injection needle (WN, Cook Medical, Bloomington, IN, USA) using a cystoscope (KarlStorz, Tuttlingen, Germany) under visual control. The MPCs were injected in the zone of the injured sphincter muscle. Comparably, sufficient sphincter deficiency was confirmed in the six gilts of cohort 3 as well on day 3 and treated with 6 × 10^5^ homologous ADSCs plus 6 × 10^5^ FITC-labelled fMPs each by two injections in the sides of the urethra as described [41]. The ADSCs were injected in the zone of the injured sphincter muscle. The regeneration of the sphincter function was monitored weekly by urodynamics for five weeks of follow-up. The mean functional gain (i.e., urodynamics “post” vs. “d35”) and the mean overall recovery (i.e., urodynamics “prä” vs. “d35”) were computed. The animals’ behaviour was observed every day to detect any health abnormalities, urine samples were collected weekly to monitor irregularities.

### 2.4. Histology of Urethral Tissue Targeted

After five weeks of follow-up, the final transurethral urodynamic measurement was performed, and the animals were sacrificed. The urethrae were prepared immediately, and the point of cell injection was ascertained by imaging of the urethra (IVIS Spectrum, Perkin Elmer, Waltham, MA, USA). The area of interest was cut out and immediately submerged in a freezing compound (TissueTec O.C.T. Sakura, Umkirch, Germany), cooled in liquid nitrogen and stored at −80 °C. Cryosections were generated (20 μm, CM1860UV, Leica, Wetzlar, Germany), and tissue was visualised by HE and AZAN staining and bright-field microscopy using a motorised sample table to scan the whole cryosections (DMi8, Leica, Supplementary Figure S1). The individual micrographs were stitched together and processed by proprietary software (LAS X, V31.0.13, Leica), exported as TIFFs, and mounted by a vector graphics program (Canvas X draw V7.0.4, CanvasGFX.com) for import in the manuscript. The fMPs were detected in the cryosections, and recorded by fluorescence microscopy (DMi8, Leica). The micrographs were processed as described above (LAS X, Leica). The fMPs were enumerated in consecutive stacks of the cryosections to determine the samples that contained the most fMPs (Figure S1). In these cryosections, the distance of the fMP clusters to the sphincter muscle, urothelial layer, and the relative positions of the fPMs in the connective tissue or submucosal layer were determined (Figure S2). The relative position of the fMPs in the urethra served as a surrogate for the localization of the injected cells and was correlated to the clinical outcome of the SUI cell therapy as gauged by urodynamics in each individual animal.

### 2.5. Power Calculation, Data Processing and Statistics

The cohort size and statistical power of the study were computed using statistical software (JMP, V17; SAS Institute Inc., Cary, NC, USA) based on the results from our preceding studies: The target variable is the urethral wall pressure in pigs after SUI induction and at different time points during follow-up. Cell-treated cohorts 2 and 3 were to be compared with the mock-treated control cohort 1. To adjust for multiple testing, the significance level had to be reduced from 5% to 5%/3 = 1.67%. Based on previous studies [40], the standard deviation of urodynamics in groups 1 to 3 was assumed to be 40 mm H_2_O. For a group difference of 80 mm H_2_O with a power of 80% to lead to a statistically significant result, five animals per group are required. If, however, one animal must be removed from the study for any reason, the total number of animals to be included in the study was increased from five to six animals per cohort.

The urodynamic measurements were conducted, and data were processed as described recently [38,40,41]. For evaluation of the results, data were imported into a spreadsheet program (Excel, version 16.93.1; Microsoft, Redmond WA, USA), sorted, and then computed by statistical software (Prism, version 10.3.1; GraphPad, Boston, MA, USA). To control the type I error rate, statistical data were adjusted with the Bonferroni correction. Statistical significance is presented as *p*-values *p* < 0.001 (***), *p* < 0.01 (**), and *p* < 0.05 (*), while *p* > 0.05 is considered not significant (n.s.).

## 3. Results

### 3.1. Spontaneous Functional Regeneration of Sphincter Deficiency

**Figure 1:**
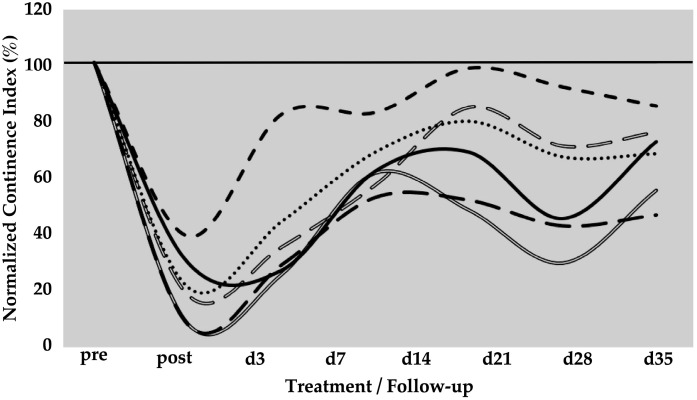
Functional recovery of the urethral sphincter complex of incontinent mock-treated control pigs. The individual lines document the urethral wall pressure of the different pigs over time. The y-axis presents the normalised urethral wall pressure (%). The x-axis denotes the time points of treatment and follow-up in days as indicated. “Pre” states the normalised wall pressure before and “post” the normalised wall pressure immediately after the induction of incontinence on day 0. For more statistical details of the urodynamics, we refer to our recent study [41].

The spontaneous regeneration of the deficient urethral sphincter muscle was monitored by urodynamics over a follow-up of five weeks (Figure 1). On day zero of the study, a significant drop of the normalised urethral wall pressure to levels of 21 ± 12% (*p* < 0.001) was recorded immediately after HF-ED and balloon dilatation when compared to the wall pressure before induction of sphincter deficiency (=100%). Spontaneous recovery without cell injection elevated the urethral wall pressure during the follow-up of five weeks to 41 ± 21% (*p* < 0.001), 64 ± 11% (*p* < 0.001), 72 ± 20% (not significant), 58 ± 23% (*p* < 0.05), and 67 ± 14% (*p* < 0.01), respectively, when compared to the urethral wall pressure immediately after surgery (i.e., “post”, Figure 1). A considerable interindividual variability between the six pigs was noted. However, the spontaneous mean recovery in the mock-control animals after five weeks of follow-up was significant (67%) when compared to the sphincter deficiency induced on day 0 (21%, *p* < 0.001; Figure 1).

### 3.2. Cell Therapy of Urinary Incontinence by Myogenic Progenitor Cells

#### 3.2.1. Therapy Efficacy of MPCs in a Porcine Animal Model of Urinary Incontinence

**Figure 2:**
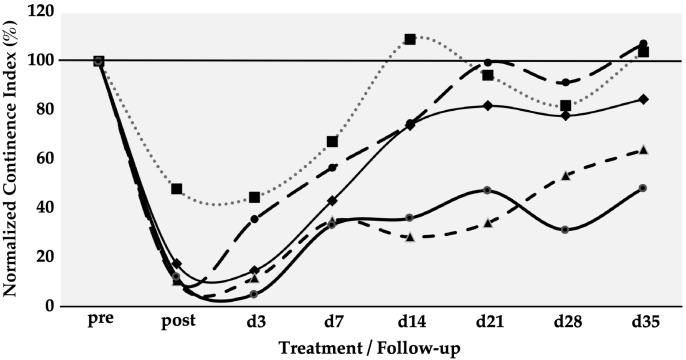
Functional recovery of the urethral sphincter complex of incontinent MPC-treated pigs. The individual lines document the urethral wall pressure of the different pigs over time. The y-axis presents the normalised urethral wall pressure (%). The x-axis denotes the time points of treatment and follow-up in days as indicated. “Pre” states the normalised wall pressure before and “post” the normalised wall pressure immediately after the induction of incontinence on day 0. For more statistical details of the urodynamics, we refer to our recent study [41].

Six gilts were treated as described above to determine the efficacy of SUI cell therapies by MPCs. The urethral wall pressure was measured before induction of sphincter deficiency and normalised to 100% in each pig (Figure 2). Immediately after induction of the sphincter deficiency, urodynamics yielded normalised wall pressure reductions to 10%, 11%, 12%, 17%, and 48%, respectively (mean 19.6 ± 16%, *p* < 0.001). This confirmed that the induction of sphincter deficiency yielded interindividually variable but significant results (Figure 2). On day 3, urodynamics revealed a spontaneous elevation of the urethral wall pressure in one of five animals before cells were injected. At the same time, in four of the five animals, the wall pressure remained at levels measured on day 0 immediately after induction of sphincter deficiency (Figure 2). However, to comply with animal welfare regulations, one pig had to be taken out of the study on day 3 before cell injection. Therefore, this cohort contained only five animals during follow-up. Then, homologous MPCs mixed with fMPCs were injected into the urethral sphincter complex of the five pigs with a Williams needle employing a cystoscope and under visual control. One week after initial surgery and four days after MPC injection, a rise in the urethral wall pressure was noted in all five MPC-treated pigs, indicating that possibly regenerative processes had occurred (Figure 2). In three of the five pigs, a further increase in the urethral muscle strength was recorded, and the MPC-mediated functional recovery continued up to 5 weeks. The pig with mild injury (56% wall pressure after SUI induction on day 0) recovered fully two weeks after initial surgery, while the sphincter recovery required more time in animals with more severe injuries (>20% wall pressure after induction). Overall, the MPC injections yielded a mean functional gain of 62% during follow-up (Table 1).

#### 3.2.2. Distribution of Particles and Cells in the Sphincter Complex of MPC-Treated Pigs After Follow-Up

**Figure 3:**
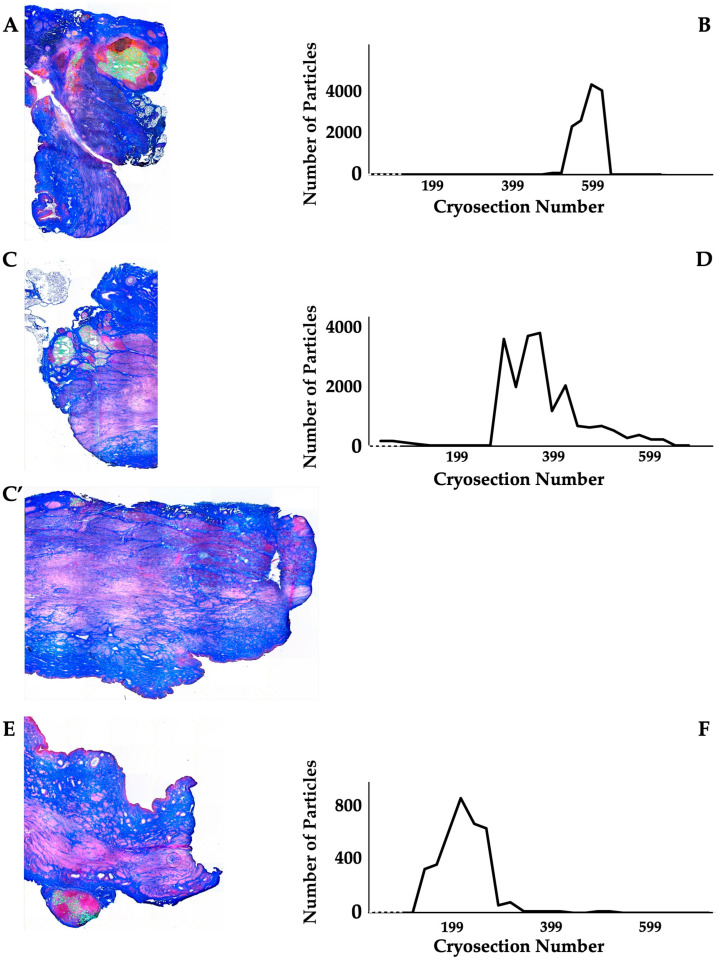
Detection of fMPs in urethral tissue samples of MPC-treated incontinent pigs #1, #2, and #5, respectively. (**A**,**C**,**C’**,**E**): Cryosections were generated and stained by AZAN to determine the urethral tissues by microscopy: Muscle tissue appears red and connective tissue blue after AZAN staining. The injected fMPs are green. (**B**,**D**,**F**): In the fluorescence mode, fMPs were detected and counted in consecutive cryosection to determine the layers with prominent signal intensities. The quantity of fMPs counted in a given cryosection is depicted on the y-axis, and the number of the corresponding cryosection is on the x-axis.

**Figure 4:**
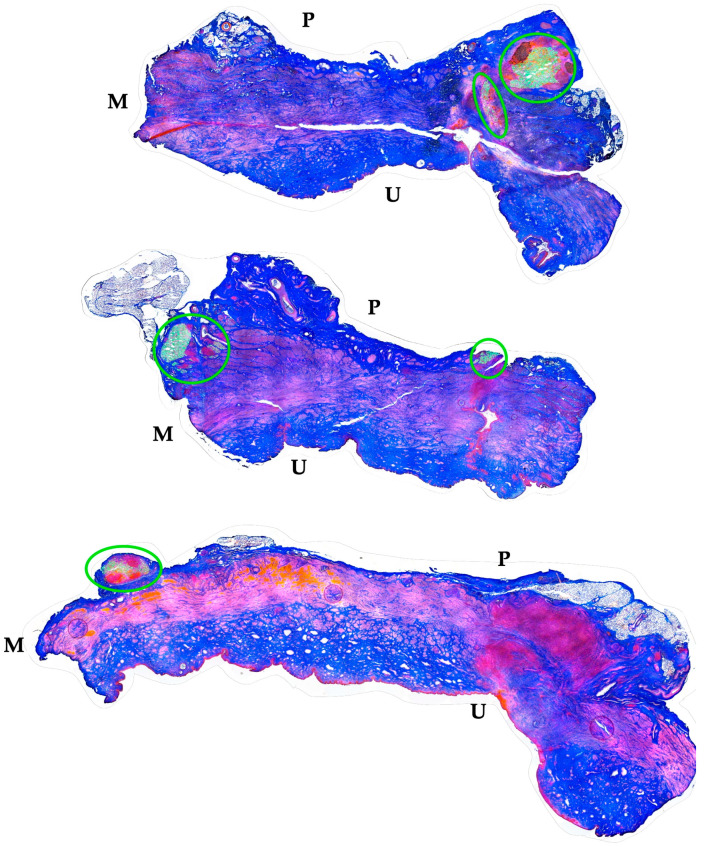
Localization of fMPs in the urethral samples of MPC-treated incontinent pigs #1, #2, and #5, respectively. The total cryosections were recorded by automated microscopy in the layer with prominent numbers of fPMs (compare Figure 3). The individual micrographs taken by transmitted light microscopy and by fluorescence microscopy were fused by an overlay mode and stitched together. “P” designates the outer peritoneal rime of the urethra, “U” the urethral lumen, and “M” the sphincter muscle in each micrograph. The connective tissue appears blue, and muscle tissue red by AZAN staining. The localisation of the injected fMPs is marked by green circles.

The distribution of MPCs and fMPs in the urethrae was recorded in three dimensions by fluorescence microscopy, and the number of fMPs was computed in the individual cryosections (Table 1; Figure S1). In animals 1 and 5, the fMPs were found clustered in a few consecutive cryosections, while the fMPs in animal 2 were distributed over several cryosections (Figure 3). The fluorescence signals recorded in the consecutive cryosections represented a sample height of approximately 2.9 mm to 5 mm (Figure 3). Then, overview micrographs were generated from the cryosections with the highest fMP counts and the positions of the injected fMPs relative to the urethral sphincter muscle, the mucosal layer, and the urothelium was investigated (Figure 4). In the MPC-treated animal 1, the fluorescent signals were observed within the urethra close to the sphincter muscle. In animal 2, both fluorescent clusters were recorded close to the sphincter muscle, still within the urethra but on the peritoneal side (Figure 4). In animal 3, fMPs were detected within the urethra, on the peritoneal side close to the sphincter muscle, while in animal 4 no particles were found [41]. In animal 5, fMPs were detected on the outer side of the sphincter muscle (Figure 4). Massive infiltrations of mononuclear cells were not observed in the zones of MPC injections (Figure 3 and Figure 4).

### 3.3. Cell Therapy of Urinary Incontinence by Adipose Tissue-Derived Mesenchymal Stromal Cells

#### 3.3.1. Therapy Efficacy of ADSCs in a Porcine Animal Model of Urinary Incontinence

**Figure 5:**
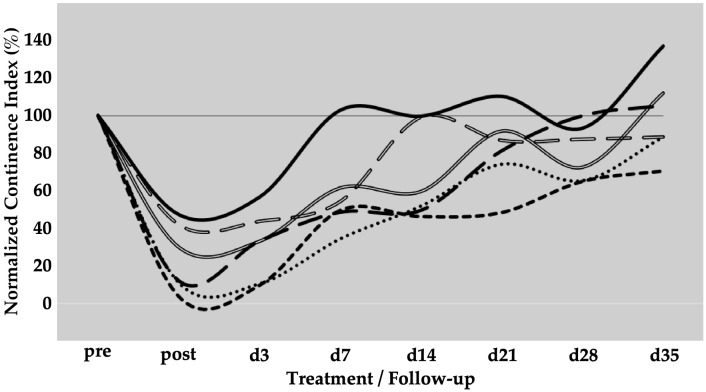
Functional recovery of the urethral sphincter complex of incontinent ADSC-treated pigs. The individual lines document the urethral wall pressure of the different pigs over time.The y-axis presents the normalised urethral wall pressure (%). The x-axis denotes the time points of treatment and follow-up in days as indicated. “Pre” states the normalised wall pressure before and “post” the normalised wall pressure immediately after the induction of incontinence on day 0. For more statistical details of the urodynamics, we refer to our recent study [41].

To determine the efficacy of SUI cell therapies by ADSCs, six gilts were treated as described above. Urodynamics on day 0, immediately after induction of sphincter deficiency, yielded reductions in urethral wall pressure in comparison to untreated animals of 4%, 11%, 12%, 30%, 42%, and 47%, respectively (mean 24.3 ± 18%, *p* < 0.001%). This confirmed that the induction of sphincter deficiency yielded interindividually variable but significant results (Figure 5). On day three, homologous ADSCs together with fMPCs were injected into the urethral sphincter complex. One week after initial surgery and four days after ADSC injection a rise in the urethral wall pressure was noted in all pigs, indicating that possibly regenerative processes had occurred (Figure 5). As observed upon MPC injections (Figure 2), pigs with a mild injury at 42% and 47% of the wall pressure after SUI induction on day 0 recovered fully to 99% and 102% of the wall pressure as early as one or two weeks of follow-up while sphincter recovery required more time in animals with more severe injuries (>25% wall pressure after induction; Figure 5). Pigs presenting with a functional loss of the urethral sphincter of approximately 90% or even 95%, recovered within five weeks of follow-up to 88% and 70% of wall pressure after ADSC injections, respectively (Figure 5). Interestingly, the slope of sphincter recovery in the individual pigs seemed less variable when compared to the overall variability of sphincter recovery in MPC-treated pigs (Figure 2). With ADSC injections, a slightly but not significantly better mean functional gain of 77% was observed during follow-up when compared to MPC injections (Table 1).

#### 3.3.2. Distribution of Particles and Cells in the Sphincter Complex of ADSC-Treated Pigs After Follow-Up

**Figure 6:**
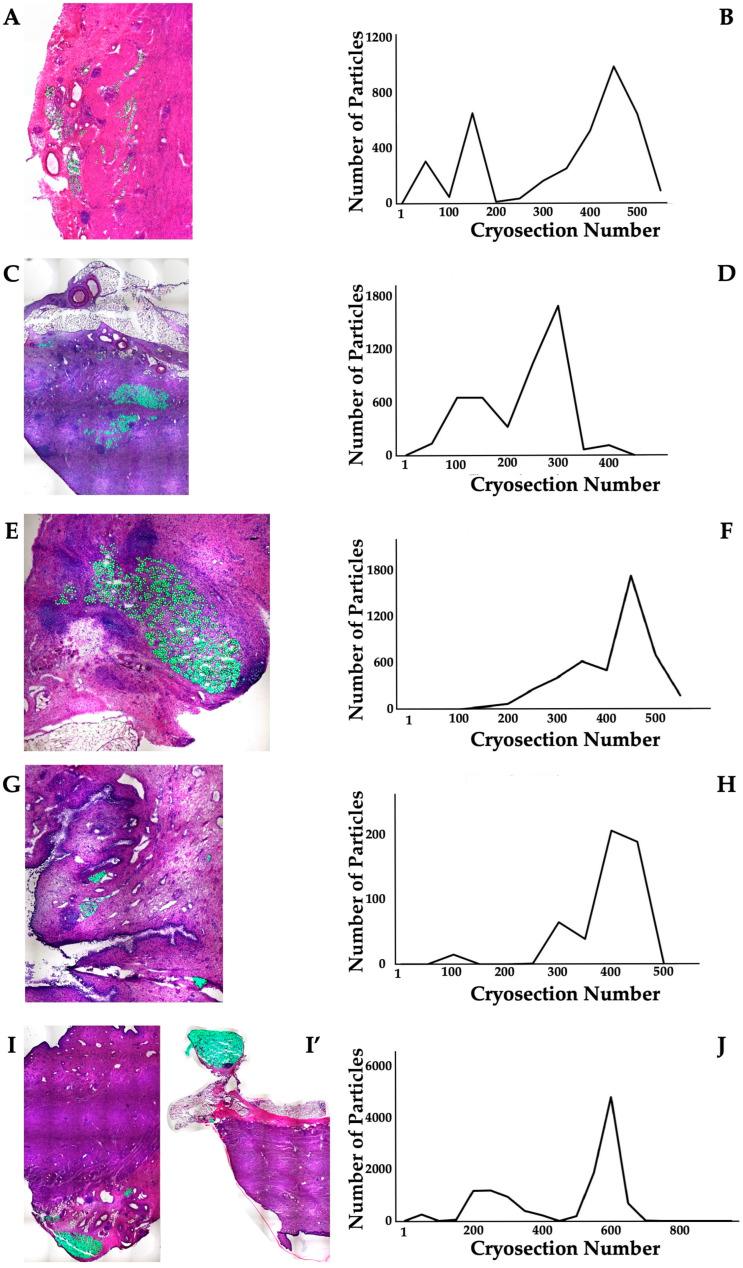
Detection of fMPs in urethral tissue samples of ADSC-treated incontinent pigs #6, #7, #8, #8’, and #9, respectively. ((**A**,**C**,**E**,**G**,**I**,**I’**); (**I**,**I’**) represent two injection sites in the same pig): Cryosections were generated and stained by AZAN to determine the urethral tissues by microscopy: Muscle tissue appears red and connective tissue blue after AZAN staining. The injected fMPs are green. (**B**,**D**,**F**,**H**,**J**): In the fluorescence mode, fMPs were detected and counted in consecutive cryosection to determine the layers with prominent signal intensities. The quantity of fMPs counted in a given cryosection is depicted on the y-axis, and the number of the corresponding cryosection is on the x-axis.

**Figure 7:**
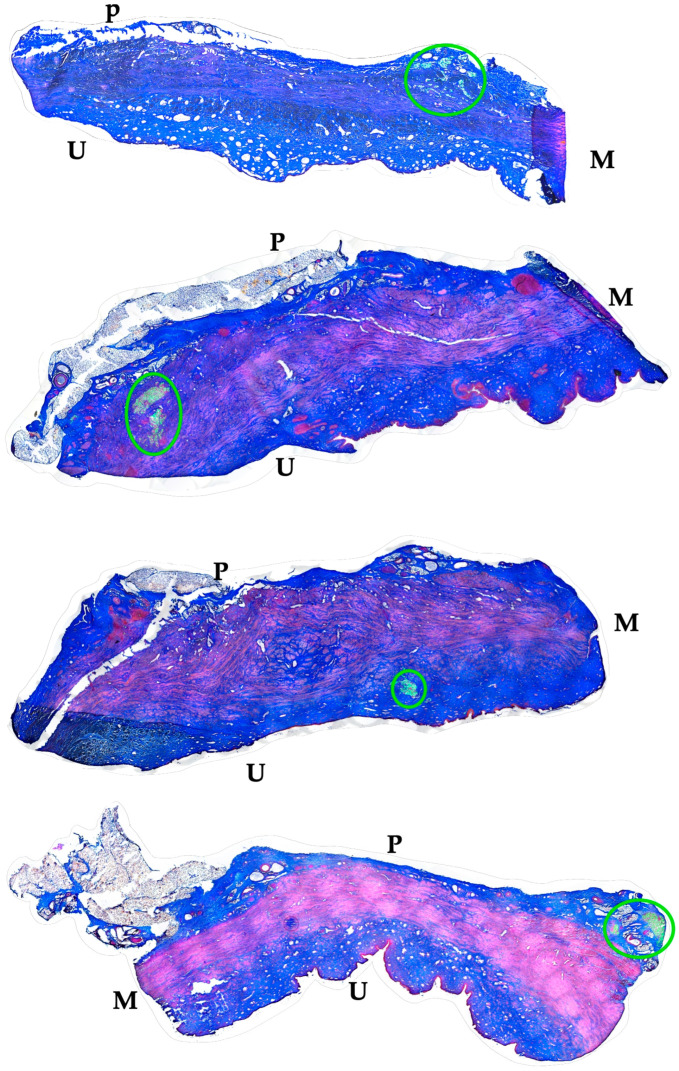
Localization of *ADSCs* in the urethral samples of *ADSC*-treated incontinent pigs #6 to #9, respectively. The total cryosections were recorded by automated microscopy in the layer with prominent numbers of fPMs (compare Figure 6). The individual micrographs taken by transmitted light microscopy and by fluorescence microscopy were fused by an overlay mode and stitched together. “P” designates the other peritoneal rime of the urethra, “U” the urethral lumen, and “M” the sphincter muscle in each micrograph. The localisation of the injected fMPs is marked by green circles.

The distribution of ADSCs and fMPs in the urethrae in three dimensions was recorded by fluorescence microscopy, and the number of fMPs was computed in the individual cryosections (Table 1; Figure S1). The distribution of fMPs as surrogates for the injected ADSCs was evaluated in consecutive cryosections of the whole tissue samples (Figure 6). In animal 6, three peaks of fMP-signals were observed stretching over more than 10 mm of cryosample’s height. Comparably, in animals 7 and 9, two distinct fMPs peaks were recorded (Figure 6). In animal 8, fluorescent signals were obtained in two distinct areas with a prominent peak each (Figure 6). The fluorescent signals observed in the ADSC-treated gilts in consecutive cryosections represented a range of approximately 4 mm to 13 mm of cryosample height (Figure 6). Then, overview micrographs were generated from the complete cryosections containing prominent fMP clusters to determine the exact zone of ADSC injection in the urethra. In animal 6, fluorescence signals indicated injection of the bulk ADSCs in the peritoneal side of the urethra but still close to the sphincter muscle (Figure 7). A different injection pattern was observed in animals 7 and 8 (Figure 7). The injected cells were detected in these two animals in the submucosal tissue of the urethra, again close to the sphincter muscle tissue (Figure 7). In animal 10, two prominent clusters of fMPs indicated successful ADSC injections in the urethra, while only a few fMPs were detected in animal 11 in any level of cryosections obtained [41]. Massive infiltrations of mononuclear cells were not observed in the zones of ADSC injections (Figure 6 and Figure 7).

### 3.4. Correlation of the Localization of Injected Cells with the Functional Recovery

The functional recovery of the induced sphincter deficiency was determined in both cohorts, the MPC and the ADSC-treated pigs (Figure 2 and Figure 5). This enabled an additional evaluation of the data by comparing the dependence of functional tissue regeneration on the location of the injected MPC or ADSCs, respectively. In the MPC-treated gilts, a full salvage of the sphincter muscle strength was achieved in animal 1 (Table 1). The injected fMP and, therefore, we assume the co-injected MPCs were localised in the submucosa of the urethra and close to the sphincter muscle (Figure 3 and Figure 4, Table 1). However, the sphincter deficiency was less prominent in animal 1 when compared to all other pigs. Thus, only a moderate functional gain of 56% was recorded (Table 1). A somewhat lower overall sphincter recovery was observed in animals 2 and 5 (Figure 2, Figure 3 and Figure 4, Table 1). In these animals, the injected fMPs, and, therefore, the co-injected MPCs, were detected in the outer rim of the urethra facing towards the peritoneum (Figure 3 and Figure 4). This position of the fluorescent signals indicated an almost full penetration of the injection needle. Of note, the induction of sphincter deficiency in these animals was prominent. Nonetheless, moderate sphincter recoveries were recorded upon MPC injections in animals 2 and 5, respectively (Table 1). Moreover, in animal 4, fluorescent signals were not observed in any of the cryosections obtained (Table 1). Thus, in this animal, full penetration of the Williams needle through the urethra injected the cells, probably in the peritoneal space [41]. At the same time, this animal yielded the weakest overall regeneration of the urethral wall pressure levels after five weeks of follow-up and gained urethral wall pressure in the range of mock-treated controls (Figure 1, Table 1). In animal 3, a functional gain of 97% and a recovery of 107% were observed (Table 1). In this animal, the fMPs and, therefore, also the MPCs had been recorded in the urethral sphincter muscle [41].

Comparably, we investigated the functional recovery of the urethral sphincter in the pig model of incontinence after injection of ADSCs as well. The best overall recovery was recorded in animals 7, 8, and 11 (Table 1). However, in animals 7 and 8, only a moderate sphincter deficiency was achieved (Figure 5). Still, a prominent functional gain of 80% and 90% was recorded after injection of ADSCs. In these two animals, prominent clusters of fMPs indicated a sufficient cell injection of ADSCs. Moreover, the fluorescence was recorded in animal 7 adjacent to the urethral sphincter muscle and in animal 8 within the submucosal tissue close to the muscle (Figure 7). As shown recently, in animal 11, a prominent fluorescence was detected on the peritoneal side of the sphincter muscle but still within the urethra [41], and in this animal, a functional gain of 83% yielded a full overall recovery of 105% (Table 1). In contrast, in animals 6 and 9, the fMPCs were recorded within the urethra but outside of the sphincter muscle (Figure 7). This correlated with a moderate overall efficacy of the cell therapy (Figure 5 and Figure 7, Table 1). In animal 10, a small cluster of fMPs indicated a less precise and efficient injection of ADSCs [41]. This correlated with a rather small functional gain and an overall weak recovery (Figure 5, Table 1).

## 4. Discussion

Loss of sphincter muscle function is regarded as a key element contributing to stress urinary incontinence (SUI) [16]. Restoration of muscular function was, therefore, considered one promising strategy to ameliorate or even cure SUI [18]. Many studies towards this goal have been based on the assumption that activation of tissue regeneration by intrinsic processes addressing, for instance, resident muscle progenitor cells [19,46] or injections of myoblasts to replace the lost and/or rebuilt sphincter muscle tissue [20,21,47,48]. Others applied mesenchymal stromal cells [33,34]. While animal studies of SUI cell therapies yielded overall very promising results [37,41], the success of clinical trials could still be improved [10,17,18,41]. Thus, we investigated the efficacy of cell therapy to treat SUI in our large animal model [40,41] employing the two most popular cell types included in these preclinical and clinical trials, MPCs and ADSCs, respectively. In our SUI pig model, sphincter deficiency was induced by a combination of HF-ED and balloon dilatation of the urethra [40,49]. When comparing the initial reduction in the sphincter function after SUI induction as determined by urodynamics, the three cohorts included in this study did not present significant differences. In mock controls, 21% of the normalised continence index was measured, and the normalised continence indices of MPC-treated (19%) and ADSC-treated gilts (24%) were not different. Thus, the initial SUI situation before cell therapy was identical to the control population. We, therefore, could compare the urodynamics after cell therapy during follow-up.

To the best of our knowledge, this study investigated for the first time the functional recovery of the urethral closure complex after cell therapy as a function of precise cell injection. At the early stage of recovery, significant elevations of the urethral wall pressure and sphincter function were not expected—not in mock-treated controls nor in animals after cell therapy. To determine the efficacy of cell therapy, a follow-up of five to six weeks seemed feasible [41]. Recent studies suggested that fluorescence-labelled cells could be detected after injection in the porcine sphincter complex for only a few days, but not after five weeks of incubation [50,51]. Moreover, direct labelling of cells by nanodots was insufficient for the efficient detection of cells in the stacks of cryosections, while direct labelling by lipophilic fluorescent dyes granted brilliant staining but modulated cell migration [52]. Moreover, lipophilic dyes inherit a high risk of unspecific labelling of the neighbouring cells and tissues [53]. Consequently, for the detection of the injection sites after a follow-up of five to six weeks, a stable label had to be co-injected to complement the cells. Preparatory studies provided evidence that fMPs could be co-injected with MPCs or ADSCs without harming their viability [41] (and unpublished data). We, therefore, assume that the co-injected fMPs marked the injection area in the urethra. In a strict sense, formal proof, e.g., by timeline studies of urethral tissue samples detecting both species, cells and fMPs, is missing and should be addressed in future studies. In addition, migration of the injected cells cannot be excluded.

On day seven of follow-up, an elevation of the urethral wall pressure was measured in all three populations. This elevation observed may include inflammatory processes, tissue swelling, and wound healing [54]. Consequently, differences between controls of cohort 1 versus cell-treated cohorts 2 and −3 were not expected, nor were significant differences between MPC- and ADSC-treated pigs anticipated early on. Of note, after five weeks of follow-up, infiltration of mononuclear cells was not observed in HE- or AZAN-stained cryosamples, nor has a robust infiltration of CD45^+^ leukocytes been recorded in representative samples by immunofluorescence [41]. This indicated that injections of cells from male littermates into female pigs did not provoke lasting inflammation. However, a trend towards enhanced muscle recovery was observed after four weeks of follow-up in MPC- or ADSC-treated pigs. In these animals, a further increase in urethral wall pressure was measured, while the urodynamics in the control cohort 1 level off. Previous experiments did not provide evidence that injected cells survived more than a few days [50,51]. Thus, it seems that the differences in sphincter recovery facilitated by MPCs or ADSCs may stem from an early induction of tissue recovery processes but yield measurable effects only in time. Here, growth factors and cytokines, vascularization, exosomes, activation of resident myogenic progenitor cells (e.g., satellite cells), and other paracrine effects come into play [19,25,27,55,56,57,58,59].

Our studies were not designed to address the question of whether MPCs differentiate and fuse to multi-nucleated myotubes that eventually integrate into the striated urethral rhabdosphincter muscle. However, this mechanism is unlikely to act in our porcine animal model. In the urethra of gilts, striated muscle tissue is found only in the very distal part [40,60], but the cells were injected into the area of the maximal urethral wall pressure, representing the sphincter complex [38,40]. It is located at more proximal sites with prominent smooth muscle tissue, known as lissosphincter muscle [40,60]. However, this notion does not exclude myotube formation from injected MPCs in other preclinical or clinical situations. Comparably, our study was not designed to investigate if the injected ADSCs differentiated into smooth muscle cells in situ. Differentiation of MSCs in smooth muscle cells was in vitro insufficient, and a stable expression of myogenic marker was not achieved [61]. We, of course, cannot exclude smooth muscle differentiation from the ADSCs, but overall, we rather hypothesise that paracrine factors promote sphincter regeneration in this SUI model.

Another aspect merits discussion as well. We observed that the precise injection of the MPCs and the ADSCs in the urethra plays a role in the clinical outcome of the sphincter regeneration. For MPCs, an injection into or very close to the lissosphincter muscle of the urethra seems preferable and yields a better clinical outcome. It is, however, not clear why MPCs, derived from a striated muscle, support the regeneration of smooth muscle tissue of the urethral sphincter complex. Unless, as outlined above, MPCs release paracrine factors facilitating tissue regeneration in more general terms, such as VEGF and angiogenin, as well as factors promoting tissue remodelling, including MMPs and their inhibitors, the TIMPs [62]. The ADSCs may release a different blend of growth factors or cytokines [27,57] and/or may survive longer in situ when compared to MPCs. Moreover, ADSCs and other MSC-like cells are known to release exosomes that promote wound healing [63]. The blend, radius of action in a given tissue, and the biological stability of ADSC-derived exosomes may contribute to the differences observed in sphincter regeneration [24,25]. Moreover, satellite cells, as well as differentiating myoblasts, have been shown to release exosomes and facilitate muscle regeneration [59,64]. The differences in the ADSC- versus MPC-derived exosomes may contribute to the differences observed in sphincter regeneration. This aspect, however, is beyond the focus of this study. Assuming that the fMP counts listed in Table 1 serve as a rough indication for the number of cells injected, a few additional notes may be added: It seems that few ADSCs produced a higher regenerative efficacy when compared to MPCs, even though the same doses of cells and fMPs was injected. Among ADSC-treated pigs, a trend to higher functional gain seems to correlate with higher fMP counts in the urethra. However, the cohort size of animals available in this study does not allow for valid conclusions. Direct labelling or quantitative detection of injected cells in the urethrae treated may yield more reliable results. But apart from that, a better effect for sphincter regeneration was achieved upon injection of ADSCs in the submucosal connective tissue close to but not into the smooth muscle.

At any rate, we must emphasise again that the cohort sizes of five or six animals limit the strength of the arguments of this feasibility study. The limitations of a porcine animal model per se are acknowledged as well. This includes differences in porcine anatomy, the limitation to healthy nullipara gilts, short follow-up timelines, and the application of cells derived from very young littermates, among others. Thus, on the way to future clinical trials, additional preclinical experimental studies are needed. These should include, for instance, dose escalation studies, timeline experiments of cell injection after sphincter injury, extended follow-up periods, and other variants of the study design (e.g., time lag between induction of incontinence and cell injection, age of the animal, nullipara versus multipara gilts), or other large animal SUI models [38,65]. The same applies to future translation of this strategy in clinical situations [10].

## 5. Conclusions

Injections of MPCs yielded a robust, and injection of ADSCs produced a significant improvement in sphincter muscle recovery within five weeks of follow-up compared to sham-treated controls in a large animal model or urinary incontinence. Three-dimensional analyses of the site of cell injection suggested that the overall functional recovery depends not only on the nature of the cells injected but is modulated in part by the precise localization of the respective cells in the tissue. The precise injection of MPCs into or very close to the urethral sphincter muscle tissue seemed to yield a higher efficacy when compared to MPC injections at distant sides. For ADSCs, the best results of urethral sphincter regeneration were achieved when the cells were placed in the submucosa of the urethra close to the sphincter muscle.

## Figures and Tables

**Table 1:** Counts of fMPs in the urethra cryosections with the largest clusters and/or high fMP density.

Animal	Cohort	Cells	fMP Counts	Functional Gain (%)	Recovery (%)
1	2	MPCs	4367	56	104
2	2	MPCs	3775	67	85
3	2	MPCs	not determined	97	107
4	2	MPCs	no fMPs found	36	48
5	2	MPCs	867	54	64
6	3	ADSCs	898	47	89
7	3	ADSCs	1664	82	112
8	3	ADSCs	1988	90	137
9	3	ADSCs	4383 *	77	88
10	3	ADSCs	not determined	66	70
11	3	ADSCs	not determined	83	105

* Plus outside urethra approx. 1437.

## Data Availability

The data are available to colleagues from Academia upon justified request.

## Supplementary Materials

The following supporting information can be downloaded at: https://www.mdpi.com/article/10.3390/biomedicines13040917/s1, Figure S1: Determination of the amounts of injected particles in paraffin sections. Clusters of injected fMPs were determined by brightfield microscopy (A). Fluorescence micrographs (B) were recorded and converted to 8-bit grayscale images (C). The background was eliminated by adjusting brightness and contrast (D,E). The particles were defined and automatically counted using the ImageJ program as described recently [52]. Size bars indicate 100 µm. Figure S2: Measuring the fMP positions in the tissue. The centre of the injected fMPs was determined in micrographs of AZAN-stained paraffin sections (black ring). The distance from the centre to the urothelial layer and urethral lumen (yellow line), to the sphincter muscle layer (orange line), or the outer rim of the urethra (green line) were determined by proprietary software programs (LAS X, V31.0.13, Leica).

## Abbreviations

ADSC	Adipose (tissue-)derived (mesenchymal) stromal cell
bFGF	Basic fibroblast growth factor
BSA	Bovine serum albumin
FBS	Fetal bovine serum
FITC	Fluorescein isothiocyanate
fMP	Fluorescent microparticle
HF-ED	High-frequency electro dissection
MMP	Matrix metalloproteinase
MPC	Myogenic progenitor cell
SUI	Stress urinary incontinence
TIMP	Tissue inhibitor of metalloproteinase
