# Supplementary material for: The Outcome of Cell Therapy Treating Urinary Incontinence Correlates with Precise Cell Localization in the Sphincter Complex
Source: Biomedicines. 2025 Apr 9;13(4):917. doi: 10.3390/biomedicines13040917 (PMC12024915; doi:10.3390/biomedicines13040917)
Supplement: Supplementary file 1 [file biomedicines-13-00917-s001.zip › biomedicines-3513258-supplementary.pdf]

Supplementary Materials:

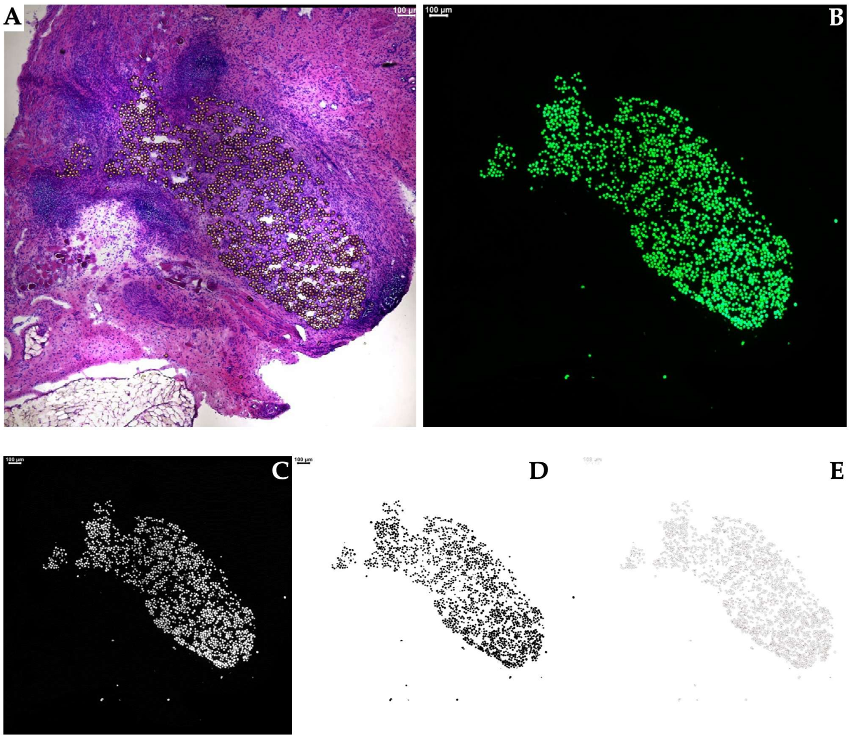

Figure S1: Determination of the amounts of injected particles in paraffin sections. Clusters of injected fMPs were determined by brightfield microscopy (A). Fluorescence micrographs (B) were recorded and converted to 8-bit grayscale images (C). The background was eliminated by adjusting brightness and contrast (D, E). The particles were defined and automatically counted using the ImageJ program as described recently (52). Size bars indicate 100 µm.

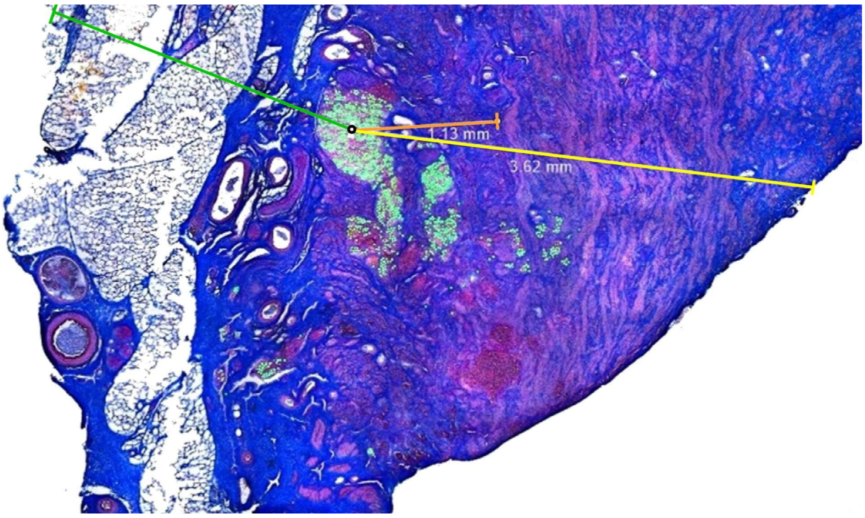

Figure S2: Measuring the fMP positions in the tissue. The centre of the injected fMPs was determined in micrographs of AZAN-stained paraffin sections (black ring). The distance from the centre to the urothelial layer and urethral lumen (yellow line), to the sphincter muscle layer (orange line), or the outer rim of the urethra (green line) were determined by proprietary software programs (LAS X, V31.0.13, Leica).
